# The Utility of Different Data Standards to Document Adverse Drug Event Symptoms and Diagnoses: Mixed Methods Study

**DOI:** 10.2196/27188

**Published:** 2021-12-10

**Authors:** Erina Chan, Serena S Small, Maeve E Wickham, Vicki Cheng, Ellen Balka, Corinne M Hohl

**Affiliations:** 1 Centre for Clinical Epidemiology and Evaluation Vancouver Coastal Health Research Institute Vancouver, BC Canada; 2 Vancouver General Hospital Pharmacy Department Vancouver, BC Canada; 3 Department of Emergency Medicine University of British Columbia Vancouver, BC Canada; 4 School of Population and Public Health University of British Columbia Vancouver, BC Canada; 5 Faculty of Pharmaceutical Sciences University of British Columbia Vancouver, BC Canada; 6 School of Communication Simon Fraser University Burnaby, BC Canada; 7 Vancouver General Hospital Emergency Department Vancouver, BC Canada

**Keywords:** adverse drug events, health information technology, data standards

## Abstract

**Background:**

Existing systems to document adverse drug events often use free text data entry, which produces nonstandardized and unstructured data that are prone to misinterpretation. Standardized terminology may improve data quality; however, it is unclear which data standard is most appropriate for documenting adverse drug event symptoms and diagnoses.

**Objective:**

This study aims to compare the utility, strengths, and weaknesses of different data standards for documenting adverse drug event symptoms and diagnoses.

**Methods:**

We performed a mixed methods substudy of a multicenter retrospective chart review. We reviewed the research records of prospectively diagnosed adverse drug events at 5 Canadian hospitals. A total of 2 pharmacy research assistants independently entered the symptoms and diagnoses for the adverse drug events using four standards: Medical Dictionary for Regulatory Activities (MedDRA), Systematized Nomenclature of Medicine (SNOMED) Clinical Terms, SNOMED Adverse Reaction (SNOMED ADR), and International Classification of Diseases (ICD) 11th Revision. Disagreements between research assistants regarding the case-specific utility of data standards were discussed until a consensus was reached. We used consensus ratings to determine the proportion of adverse drug events covered by a data standard and coded and analyzed field notes from the consensus sessions.

**Results:**

We reviewed 573 adverse drug events and found that MedDRA and ICD-11 had excellent coverage of adverse drug event symptoms and diagnoses. MedDRA had the highest number of matches between the research assistants, whereas ICD-11 had the fewest. SNOMED ADR had the lowest proportion of adverse drug event coverage. The research assistants were most likely to encounter terminological challenges with SNOMED ADR and usability challenges with ICD-11, whereas least likely to encounter challenges with MedDRA.

**Conclusions:**

Usability, comprehensiveness, and accuracy are important features of data standards for documenting adverse drug event symptoms and diagnoses. On the basis of our results, we recommend the use of MedDRA.

## Introduction

### Background

Adverse drug events are the harmful and unintended consequences of medication use and are a leading cause of emergency department visits and hospitalizations in Canada and internationally [1-4]. Adverse drug events comprise various types of medication-related problems, including adverse drug reactions (ie, noxious effects that occur within a standard dosing range of a prescription drug). Adverse drug events frequently recur without documentation and communication, which compromises patient safety [5]. The incidence, severity, and recurrence of adverse drug events suggest a need for greater documentation and communication of such events to avoid patients being re-exposed to harmful medications [5].

Adverse drug event reporting is voluntary for clinicians in Canada but has recently become mandatory for hospitals [6]. The implementation of mandatory reporting for hospitals introduces concerns about the added burden of documentation for clinicians. There is often a disconnect between adverse drug event reporting and clinical care activities because of time constraints and a poor fit between standardized nomenclatures built into inflexibly designed reporting systems [7]. The existing electronic medical records include data fields for documenting allergies but can be restrictive and inappropriate for documenting adverse drug reactions and other types of adverse drug events [7]. Furthermore, allergies and adverse drug reactions are a fraction of all the reportable clinically significant adverse drug events [5]. Even when broader input fields are available to document adverse drug events, they are often in free text format, and thus, the resulting data are unstructured, nonstandardized, and prone to misinterpretation. As a result, clinicians who diagnose and treat adverse drug events rarely report them in the existing electronic systems [1,4,8]. Enabling data entry using standardized terminology may reduce the ambiguity of adverse drug event reports, ease the data entry process, improve the utility of the systems, and thereby improve patient safety [9,10]. However, the use of standardized data systems that are incompatible with clinical work may also compromise patient safety and reduce the quality and availability of data for research purposes [11].

### Objective

System designers may leverage a number of existing national and international standards to support documentation; however, few studies have examined which data standard is preferable for capturing details about adverse drug events. This study aims to understand and compare the utility of different clinical data standards in capturing adverse drug event symptoms and diagnoses. This has been undertaken in relation to a new law in Canada that mandates the reporting of serious adverse drug reactions [6]. We hope to provide insight into the strengths and weaknesses of different standards as vendors begin to develop software to support adverse drug event documentation.

## Methods

### Study Design

This was a mixed methods substudy of a multicenter retrospective chart review [5]. We used a convergent mixed methods design in which we collected and analyzed quantitative and qualitative data concurrently and separately and then merged and compared them during the interpretation phase [12].

### Setting and Population

We reviewed the research records of all patients who were diagnosed with ≥1 adverse drug event in 1 of the 3 prospective multicenter studies [13-16]. The first study enrolled 1591 patients presenting to the emergency departments of 2 tertiary care hospitals—Vancouver General Hospital (VGH) and St Paul’s Hospital—in Vancouver, British Columbia, Canada, from 2008 to 2009 and derived a clinical decision rule to identify the patients at high risk of adverse drug events [13]. The second study enrolled 10,807 patients presenting to the emergency departments of VGH, Lions Gate Hospital (an urban community hospital in North Vancouver, British Columbia), and Richmond Hospital (an urban community hospital in Richmond, British Columbia) between 2011 and 2013 and evaluated the impact of a pharmacist-led medication review on health outcomes [14,16]. The third study enrolled 1529 patients presenting to the emergency departments of VGH, Lions Gate Hospital, and the Ottawa Civic Hospital (an urban tertiary care hospital in Ottawa, Ontario, Canada) from 2014 to 2015 and validated the previously derived clinical decision rule [15].

In all 3 prior studies, the research assistants used a systematic selection algorithm to select and enroll a representative sample of emergency department patients (Multimedia Appendix 1). A clinical pharmacist and physician evaluated all the enrolled patients with adverse drug events at the point of care and documented the events in research and medical records. All the cases in which the clinical pharmacist diagnoses and physician diagnoses were concordant were considered final. An independent committee adjudicated all the cases in which the assessments were discordant or uncertain by reviewing the research and medical records.

### Inclusion and Exclusion Criteria

This study included all adverse drug events that met our case definition and were diagnosed in 1 of the 3 primary studies (see *Case Definition* section). We excluded events with alternative diagnoses, those for which records could not be retrieved or were illegible, and those that were not unique with respect to the drug and presenting symptom or diagnosis [13-16].

### Case Definition

Adverse drug events included adverse drug reactions, drug interactions, supratherapeutic or subtherapeutic dosing, untreated indications, drug withdrawal, ineffective drugs, nonadherence, and errors in prescribing, dispensing, or medication administration [5,13-16]. These adverse drug events had to be classified as moderate, resulting in a change in medical management, diagnostic testing, or consulting or severe, resulting in hospital admission, permanent disability, or death [5,16].

### Chart Review Data Collection Methods

A total of 2 research assistants (EC, a clinical pharmacist and VC, a pharmacy student) retrospectively reviewed the research records of the enrolled patients. They were independent and blinded to one another’s data collection and applied the different data standards to document up to 4 symptoms or diagnoses that they felt were appropriate to describe each adverse drug event using an electronic data collection form (Multimedia Appendix 2). If the research assistants were unable to identify an appropriate symptom or diagnosis, they selected *No Match*. The research assistants then documented whether they thought the terms selected in the data standard accurately described the case.

We conducted a pilot period to ensure the quality of the data collected and identify any potential questions about the application of our research protocol. During the pilot period, the research assistants collected data on a sample of 20 adverse drug events and subsequently provided feedback on the data collection form. We edited the form following the provision of feedback, which the research assistants then piloted on a new sample of 20 records, resulting in a total of 40 records being piloted.

During the pilot period, the research assistants met weekly to discuss discordant cases in which there were disagreements in the identified symptoms or diagnoses for each data standard to ensure consistency in case interpretation. After the pilot period, the research assistants met monthly to discuss the discordant cases in which there was disagreement in the accuracy of the data standard in describing the case. We considered all cases in which the research assistants reached a consensus on the various data standards as final (Multimedia Appendix 3).

We randomly selected 100 adverse drug events for the research assistants to assess twice to evaluate intrarater reliability.

### Qualitative Data Collection Methods

During the chart review, the research assistants electronically recorded notes on their process, general impressions, and any case-specific challenges they encountered for each data standard. A qualitative researcher (SS) attended the meetings between the research assistants to observe the discussion of the discordant cases and took notes on the discussion to capture emerging themes and points of convergence and divergence. This produced a richer understanding of the human factors that influence the perceived utility of data standards.

### Data Standards

We used four data standards to document the symptoms and diagnoses of each adverse drug event: Systematized Nomenclature of Medicine (SNOMED) Health Concern and Diagnosis (SNOMED HC) reference set, SNOMED Adverse Reaction (SNOMED ADR) reference set, Medical Dictionary for Regulatory Activities (MedDRA), and International Classification of Diseases (ICD) 11th Revision. We selected these as various levels of government and other organizations in Canada recommend their use in different clinical contexts related to adverse drug event reporting and documentation.

#### SNOMED HC Reference Set

SNOMED Clinical Terms (SNOMED CT) is an international clinical terminology coding system that includes diagnoses, signs, symptoms, and diagnostic procedures. We used the SNOMED CT Canadian Edition, which was developed specifically for use in Canada and released in October 2018 [17]. The SNOMED HC reference set is a subset of SNOMED CT, which is designed to map terminology to the ICD-9 and ICD-10 codes and the Canadian Emergency Department Diagnosis Shortlist for use in electronic medical records and clinical information systems. We included SNOMED CT as it is maintained and recommended by Canada Health Infoway to support the capture and exchange of clinical data in Canada [18].

#### SNOMED ADR Reference Set

The SNOMED ADR reference set highlights the allergies and intolerances found in SNOMED CT. This is a baseline reference set under development, which will continue to expand based on feedback. The Northern Health Authority in British Columbia has integrated this data standard into their electronic medical record system. Users select the terminology through a search function, which creates a filtered dropdown selection list. We included SNOMED ADR as it is under development for use specifically in British Columbia [19].

#### MedDRA Preferred Terms

MedDRA version 22.0 is an international standardized medical terminology dictionary that supports classification of adverse event information associated with biopharmaceuticals and other medical products [20]. MedDRA’s hierarchical structure comprises 5 terminological levels that map to one another, arranged from very specific to very general. We used the *Preferred Terms* level, which presents terms as distinct descriptors for symptoms, signs, disease diagnoses, therapeutic indications, investigations, surgical or medical procedures, and medical, social, or family history characteristics. We modified some terms from their original British spelling to their American spelling by referring to corresponding Lower Level Terms in the MedDRA hierarchy. We included MedDRA as its use is recommended by Health Canada for adverse reaction reports submitted to their pharmacovigilance database [21].

#### International Classification of Diseases 11th Revision

ICD-11 is an international standard for reporting diseases and health conditions for clinical and research purposes [22]. The World Health Organization released the 11th revision in 2018 for piloting, and it will come into use in 2022. This version provides a coding system designed for easy adoption into electronic environments and updated clinical content, including explicit coding to capture adverse drug events. We included ICD-11 as it is used by physicians in British Columbia for claims submissions to the provincial Medical Services Plan [23].

### Quantitative Analysis

We used descriptive statistics to describe the baseline characteristics of all included adverse drug events using proportions. To determine the coverage of a specific data standard to capture an event, we used the research assistants’ ratings of whether a data standard contained a match for the characteristics of an adverse drug event. We used the consensus assessments to calculate the frequency and proportion of adverse drug events with symptoms or diagnoses found within a given data standard.

We allowed entries for up to 4 terms per data standard. To identify whether the research assistants agreed on the term selected within a given data standard, we examined whether the first selected term for each data standard matched (first term match). Then, we examined whether there were any matches across the 4 terms between the terms used by both research assistants for each data standard (all terms match).

### Qualitative Analysis

We coded comment fields from the data collection forms and notes from our observations using NVivo (version 12; QSR International) qualitative data analysis software. We began by inductively coding field notes to generate a provisional coding structure that we then applied to the comment fields. We iteratively reviewed the data and coding to identify emerging themes. We completed an interim review of the coding and emerging findings to contextualize the results with quantitative data and validate them with the research assistants’ experiences. Following discussion, we generated a final coding structure and used a descriptive approach to describe the classification challenges.

## Results

### Quantitative Results

**Figure 1:**
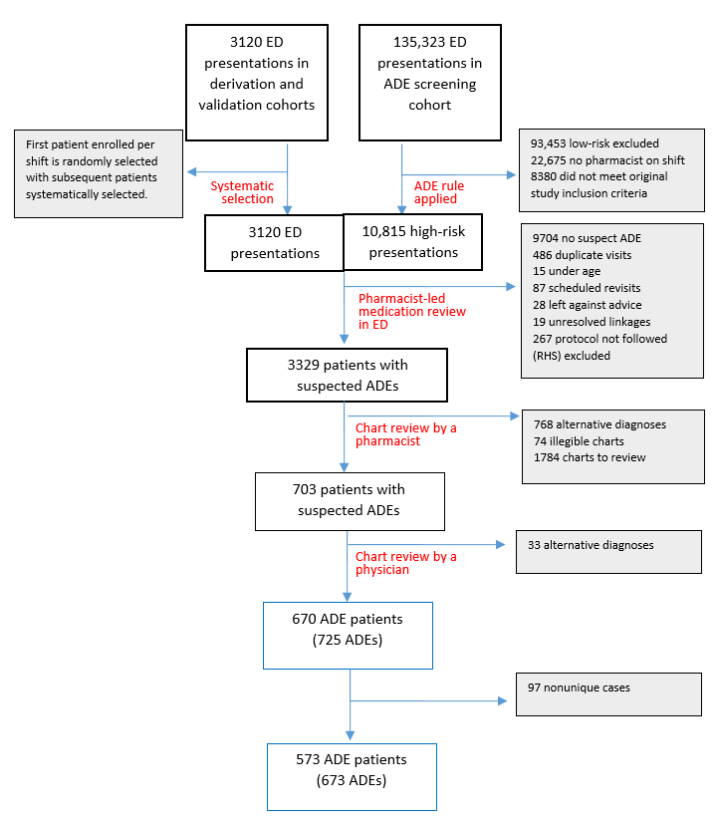
Flow diagram of patients through the study. ED: emergency department; ADE: adverse drug event.

Figure 1 displays the flow of patients in the study sample. Overall, we included 673 adverse drug events in 573 patients in our sample. The top 5 most common culprit drugs and diagnoses of the included adverse drug events are presented in Table 1. The most common culprit medication overall was warfarin (62/673, 9.2%, 95% CI 7.1%-11.7%), and the most common diagnosis was allergic reaction (38/673, 5.7%, 95% CI 4%-7.7%).

Table 2 displays the coverage of the different data standards for the adverse drug events in our sample. Of the data standards, MedDRA (671/673, 99.7%, 95% CI 98.9%-99.9%) and ICD-11 (667/673, 99.1%, 95% CI 98%-99.6%) had the highest frequency of having an appropriate symptom or diagnosis available. SNOMED ADR had the lowest frequency (576/672, 85.7%, 95% CI 82.7%-88%).

**Table 1:** Adverse drug event (ADE) characteristics of the study sample (N=673).

Characteristics		ADEs, n (%)
**Culprit medication**		
	Warfarin	62 (9.2)
	Furosemide	37 (5.5)
	Acetylsalicylic acid	37 (5.5)
	Hydrochlorothiazide	26 (3.9)
	Insulin	21 (3.1)
**ADE diagnosis**		
	Allergic reaction	38 (5.7)
	Laboratory abnormality	36 (5.4)
	Stroke	22 (3.3)
	Hypoglycemia	20 (3)
	Atrial fibrillation	19 (2.8)

**Table 2:** The percentage of having an appropriate symptom or diagnosis available to describe the adverse drug event cases by data standard (N=673).

Data standard	Symptom or diagnosis option available, n (%, 95% CI)
MedDRA^a^	671 (99.7, 98.9-99.9)
SNOMED ADR^b,c^	576 (85.7, 82.7-88)
SNOMED HC^d^	650 (96.6, 94.9-97.79)
ICD-11^e^	667 (99.1, 98-99.6)

^a^MedDRA: Medical Dictionary for Regulatory Activities.

^b^SNOMED ADR: Systematized Nomenclature of Medicine Adverse Reaction.

^c^N=672.

^d^SNOMED HC: Systematized Nomenclature of Medicine Health Concern and Diagnosis.

^e^ICD-11: International Classification of Diseases 11th Revision.

Table 3 presents the percentage agreement between the research assistants for the first term match and for any term match for each data standard. For the first term match, SNOMED HC (409/673, 60.8%, 95% CI 57%-64%) yielded the most matches between the research assistants and ICD-11 (286/673, 42.5%, 95% CI 38.8%-46.3%) yielded the fewest matches. In terms of having any term match, MedDRA performed the best (673/673, 100%, 95% CI 99.4%-100%) and ICD-11 yielded the lowest proportion of matches (583/673, 86.6%, 95% CI 83.9%-89%). Semantic differences between terms with identical meanings within a data standard may have artificially lowered the number of matches for a given data standard. For example, in SNOMED HC, 1 research assistant selected the term *frank hematuria*, whereas the second research assistant selected the term *blood in urine* to describe hematuria. In SNOMED ADR, 1 research assistant selected *muscle weakness*, and the second selected *asthenia* to describe weakness. In ICD-11, 1 research assistant selected *candidiasis of lips or oral mucous membranes*, and the second selected *thrush disorder* to describe thrush. The complete list of adverse drug events that did not have a match on any of the data standard terms is presented in Multimedia Appendix 4.

**Table 3:** The percentage agreement between the research assistants in coding the adverse drug events by data standards (N=673).

Data standard	First term match, n (%)	Any terms match, n (%)
MedDRA^a^	400 (59.4)	673 (100)
SNOMED ADR^b^	379 (56.3)	601 (89.3)
SNOMED HC^c^	409 (60.8)	629 (93.5)
ICD-11^d^	286 (42.5)	583 (86.6)

^a^MedDRA: Medical Dictionary for Regulatory Activities.

^b^SNOMED ADR: Systematized Nomenclature of Medicine Adverse Reaction.

^c^SNOMED HC: Systematized Nomenclature of Medicine Health Concern and Diagnosis.

^d^ICD-11: International Classification of Diseases 11th Revision.

### Qualitative Results

**Figure 2:**
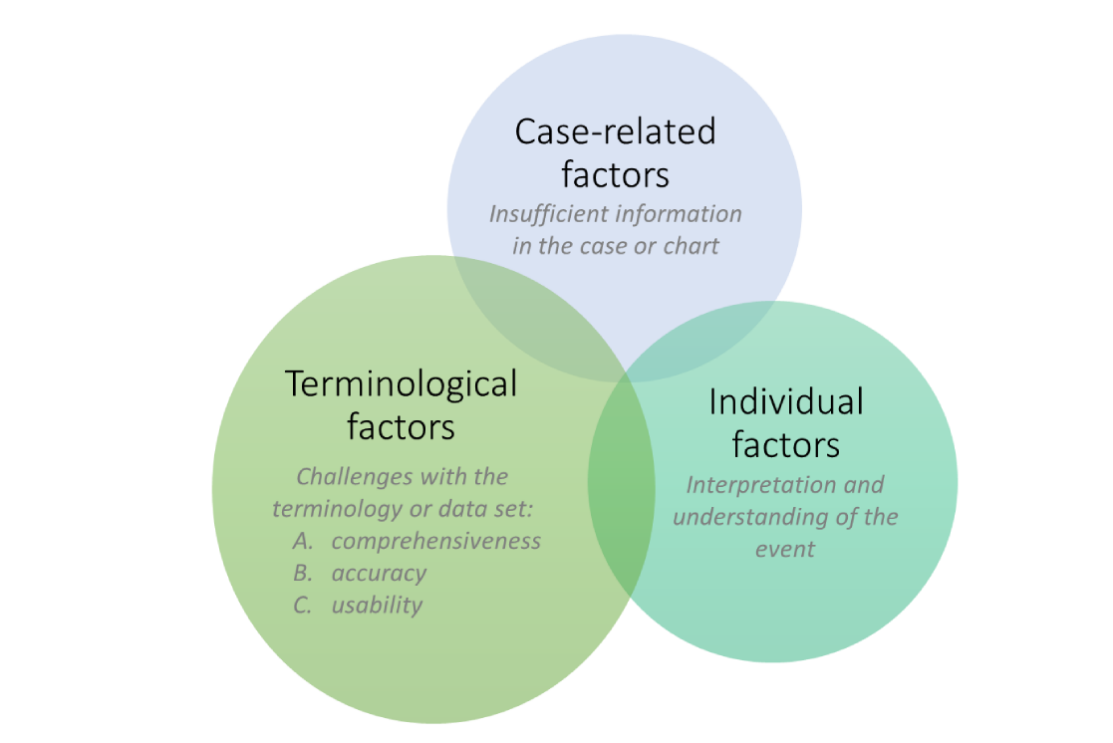
Classification challenges in qualitative analysis.

We found 3 primary factors that affected the classification of adverse drug event symptoms and diagnoses: (1) terminological factors specific to the terminology or data standard, (2) case-related factors in which there was not enough information in the patient’s chart to classify the event appropriately, and (3) individual factors related to interpretation and recall (Figure 2).

#### Terminological Factors

Terminological challenges were the most common factors that affected the adverse drug event classification. We assessed the overall utility of each data standard according to 3 key terminological factors: comprehensiveness (conceptual coverage or breadth; eg, is the data source comprehensive enough to select appropriate terms?), accuracy (terminological correctness or exactness; eg, does the available terminology accurately describe the case?), and usability (ease of use; eg, is it easy to find an appropriate term using the data source?).

We used the ability to locate any term to describe the main symptom or diagnosis as a proxy variable for comprehensiveness. For example, both research assistants noted that the primary symptom and diagnosis of an adverse drug event were *hemiparesis* and *stroke*; however, there was no option to describe this case in SNOMED ADR. Poor comprehensiveness was found most often when using SNOMED ADR and rarely when using MedDRA, which is consistent with our quantitative findings related to coverage.

Issues with the accuracy of data sources emerged when the terms did not fully capture or represent the case, were too specific, or were too vague. In the case of the main adverse drug event symptom or diagnosis being *fall*, the research assistant found partial terminology in SNOMED ADR, including *weakness* and *syncope and collapse*; however, during consensus, the research assistant noted that the patient did not have syncope. In this case, although some terms were available, they did not produce a complete and clinically meaningful or accurate description of the event. An instance in which the terminology was too specific arose with ICD-11, wherein the only term available to describe a hematoma case included the qualifier *of other specified site complicating a procedure*; however, there was no indication that this hematoma was in fact complicating a procedure. An example of exceedingly broad or vague terminology arose when the research assistants could not find a specific term for *Clostridium difficile* in SNOMED ADR, which led to a discussion of whether the term *diarrhea* was adequate. These challenges occurred most often with SNOMED ADR and least often with MedDRA.

Unusual terminology and phrasing or unfamiliar spelling compromised the usability of the data sources. Usability challenges emerged when the research assistants reported that they felt a term adequately described the case but that it was difficult to find, it was only identified during consensus, or they had to rely on external sources to identify the term (eg, Google). In the case of a *rash*, the research assistants identified the term *allergic disorder of the skin* in ICD-11 as the closest descriptor but felt that it was atypical phrasing. Another issue was the use of British English spellings for certain terms (eg, *haemorrhage* instead of *hemorrhage*). Usability challenges were most common with ICD-11; however, the research assistants encountered them less often than the issues with accuracy or comprehensiveness.

#### Case-Related Factors

In some cases, there was insufficient information in the chart to classify the event independent of the data source. For example, in a case, the research assistant noted that the selected term was broad enough, but the classification would have been improved if there were more details about the patient’s documented *bizarre behavior*. The research assistants also encountered cases with insufficient information to classify the event in the context of the data standard’s limitations, often because of vague case descriptions that could only be classified using high specificity terms. For example, in SNOMED ADR, the terms to describe headaches were often too specific, such as *frontal headache* or *migraine with aura*, whereas case descriptions tended to use only the term *headache*.

#### Individual Factors

Individual factors, such as recall and interpretation, had an effect on the classification of events. During consensus, the research assistants discussed instances where 1 research assistant did not identify the correct term that the other had identified. This occurred because they forgot the terminology (eg, the research assistant had been searching for the term *kidney* rather than *renal* to describe abnormal renal function), did not consider alternate wording (eg, the research assistant did not think of the term *spasticity* to refer to *rigidity* or *stiffness*), or were unable to locate a term that they felt was acceptable (eg, the research assistant could not find a term related to the patient’s history of noncompliance). In almost all of these instances, the research assistant agreed with the other’s selection during the consensus.

## Discussion

### Principal Findings

Previous studies have demonstrated gaps in the existing terminological standards in health care [24,25]. Our findings add a nuanced examination of these gaps and other shortcomings of multiple terminological standards rooted in clinical practice. We explored the utility of 4 data standards to document adverse drug event symptoms and diagnoses. Our quantitative analysis demonstrated that MedDRA and ICD-11 were most likely to have an appropriate symptom or diagnosis available. MedDRA most often had any match documented, whereas ICD-11 had the fewest matches. SNOMED HC performed the best in terms of the first term that the research assistants selected for matching. SNOMED ADR performed the worst in terms of having the lowest capture of a symptom or diagnosis. These results are consistent with our qualitative findings. The research assistants were least likely to encounter terminological challenges with MedDRA and most likely with SNOMED ADR. We found that ICD-11 was most likely to present usability challenges because of unusual terminology or spelling, which may provide a rationale for why ICD-11 had the fewest matches. The research assistants also found ICD-11 to be the most time consuming for searching terms because of the lengthy list of returned matches with descriptive terms, whereas SNOMED HC and SNOMED ADR were the least time consuming, with a shorter list of returned matches with more straightforward terms to select from. Overall, across all the indicators, we found that MedDRA was the strongest data standard, whereas SNOMED ADR performed poorest. We acknowledge that SNOMED ADR is a working data set at this time and thus could be strengthened through further study and use.

Implementing clinical information systems with data standards that lack comprehensiveness, accuracy, or usability in clinical practice will affect data entry and generate downstream negative effects on data quality and the information generated. In the absence of correct or accurate terminology, research assistants were more likely to make compromises or use workarounds by selecting a term that was close enough or only partially described the event. In clinical practice, challenges with data entry, along with time constraints and other external pressures, may result in a clinician opting to abandon data entry altogether, thus lowering data quantity and quality. Conversely, semantic standardization may lead to more consistent and complete reporting for pharmacovigilance activities if the appropriate data standard is used, which may produce higher data quality and facilitate data analysis [26-28]. Reliable coding for adverse drug reactions is likely to yield more meaningful data for the end user and may facilitate data integration across different electronic health systems [28,29].

Recent efforts to map terminology across different standards may be used to develop clinical information systems with specific data standards while facilitating data integration across systems and pharmacovigilance organizations. Reich et al [30] demonstrated that it is feasible to map ICD-9 diagnosis codes for medical conditions to SNOMED CT and MedDRA, making both suitable options for standard vocabularies. However, to our knowledge, there has been no study that has compared these data sets with one another or investigated their use specifically for documenting adverse drug events [30]. The WEB-RADR 2 Project seeks to develop a bidirectional mapping of a subset of pharmacovigilance terms between SNOMED CT and MedDRA [31]. Mapping and testing were scheduled to be completed in 2020, with a production version of the map available to SNOMED and MedDRA users in 2021. In addition, the National Institutes of Health’s National Library of Medicine has also developed a Unified Medical Language System that maps SNOMED CT to ICD-10 to support reimbursement and statistical analyses [32]. Further research should examine the effect of mapping data standards on data quality.

In addition to the efforts to map terminology across data standards, advances in natural language processing increasingly offer a new and promising approach to the analysis of adverse drug event reports for pharmacovigilance activities. Using natural language processing, system designers may enable free text data entry from clinicians to increase the ease of use. Such systems would then algorithmically analyze the entered data to produce standardized data for monitoring and regulatory purposes [33]. A recent systematic review found that many studies on natural language processing of incidents, adverse events, and medical error reports have focused primarily on binary classification, which does not account for the complexity of adverse drug event documentation that we sought to capture and limits the subsequent clinical utility of data to support continuity of care [33]. In addition, in instances where cases contain insufficient information for classification, which we encountered in this study, natural language processing is unlikely to improve the results. Continued research in this field should explore natural language processing, aim to produce multimodal analyses of reports, and increase integration across clinical information systems.

In the absence of an agreed-upon standard for data capture and with the advent of increased mapping across terminologies, we suggest that health system designers prioritize the implementation of a data standard that is clinically useful and relevant to ensure high usability for clinicians who are asked to document the event while being immersed in clinical activities. For this purpose, MedDRA was the strongest data standard among the data sets in our study, and we recommend it be used as the standard for Canadian pharmacovigilance activities in support of federal legislation that requires all Canadian health institutions to report serious adverse drug reactions to Health Canada [6]. MedDRA is also currently used in other pharmacovigilance systems, such as the US Food and Drug Administration Adverse Drug Event Reporting System and Vaccine Adverse Event Reporting System databases, the European Medicines Agency Eudrawatch system, and the Japanese prescription event monitoring system, which makes it a strong option to advance international collaborative efforts in pharmacovigilance.

### Limitations

There are limitations to this study. Our research team is more familiar with MedDRA, which may have led to bias when selecting the most comprehensive data standard. However, one of our research assistants had previously never worked with our team and thus was unfamiliar with MedDRA at the outset of this study. We also relied on the American translation of British MedDRA terminology, which may have facilitated the identification of terms and resulted in more matches. The ICD-11 terms remained in their original British spelling, which may have initially resulted in fewer matches. We observed that this effect was offset as the research assistants became familiar with these patterns over time and with use. SNOMED ADR is designed to describe adverse drug reactions; however, we applied it to describe a broader range of adverse drug events. This may have positioned SNOMED ADR to perform poorly from the outset and falsely lowered its capture of a symptom or diagnosis compared with the other data standards. We were also unable to obtain more information than what was available in the research records, as they were from a previous multicenter chart review study, which may have limited the terms we could have selected for a given data standard.

### Conclusions

Usability, comprehensiveness, and accuracy are the key features of a data standard for documenting adverse drug event symptoms and diagnoses. On the basis of these factors, we found that MedDRA is the most suitable data standard for coding adverse drug events in electronic reporting systems. Although data standardization is important, not all standards are created equally. As our analyses demonstrate, each data standard has different affordances and constraints. Hence, it is important to critically evaluate competing standards to ensure that the data standards adopted in clinical information systems support patient safety rather than compromise it. When the appropriate data standard is selected, the standardized terminology may result in more consistent adverse drug event documentation and better data quality and quantity as a by-product of routine care.

## Appendix

Multimedia Appendix 1 Patient enrollment algorithm.

Multimedia Appendix 2 Data collection form.

Multimedia Appendix 3 Consensus data collection form.

Multimedia Appendix 4 Adverse drug events with no matching terms.

## Abbreviations

ICD: International Classification of Diseases
MedDRA: Medical Dictionary for Regulatory Activities
SNOMED: Systematized Nomenclature of Medicine
SNOMED ADR: Systematized Nomenclature of Medicine Adverse Reaction
SNOMED CT: Systematized Nomenclature of Medicine Clinical Terms
SNOMED HC: Systematized Nomenclature of Medicine Health Concern and Diagnosis
VGH: Vancouver General Hospital
