# Supplementary material for: The Utility of Different Data Standards to Document Adverse Drug Event Symptoms and Diagnoses: Mixed Methods Study
Source: J Med Internet Res. 2021 Dec 10;23(12):e27188. doi: 10.2196/27188 (PMC8709916; doi:10.2196/27188)
Supplement: Multimedia Appendix 1 [file jmir_v23i12e27188_app1.docx]

## Appendix 1. Patient Enrollment Algorithm.

*Time -60 to 0 min*

Patients presented to the ED in a random sequence in the hour prior to the start of each data collection shift.

Start of data collection shift.

*Time 0*

Research assistant tallied the total number of patients (n) who presented between -60 min and Time 0, and entered n into a computerized random number generator.

The computer produced a random number (x) between 1 and n.

The research assistant enrolled the x^th^ patient who presented between -60 min and Time 0. If this patient was not eligible, the next eligible patient was enrolled.

*Time 0 +45min*

After the assessment of the first enrolled patient, the patient who presented 45 min after the first enrolled patient was approached. If this patient was not eligible, the next eligible patient was approached.

After the assessment of the first enrolled patient, the patient who presented 45 min after the first enrolled patient was approached. If this patient was not eligible, the next eligible patient was approached.

*Time 0 +90min*

Chart Review by a pharmacist

Once finished with the first enrolled patient, the research assistant enrolled consecutive eligible patients presenting in 45 min intervals after the last enrolled patient.
