# Supplementary material for: The Utility of Different Data Standards to Document Adverse Drug Event Symptoms and Diagnoses: Mixed Methods Study
Source: J Med Internet Res. 2021 Dec 10;23(12):e27188. doi: 10.2196/27188 (PMC8709916; doi:10.2196/27188)
Supplement: Multimedia Appendix 2 [file jmir_v23i12e27188_app2.docx]

## Appendix 2. Data collection form.

## Data Collection Form

**ADE Information**

| Record ID | Automatically assigned by REDCap (numbered 1-573) |
| --- | --- |
| ADE Information | |
| ADE ID | *Preloaded from dropdown list of ADE cases for review* |
| ADE Drug 1 | *Preloaded from dropdown list by ADE case* |
| ADE Drug 2 | *Preloaded from dropdown list by ADE case* |
| ADE Drug 3 | *Preloaded from dropdown list by ADE case* |
| Other ADE Drug 1 | *Preloaded from dropdown list by ADE case* |
| Other ADE Drug 2 | *Preloaded from dropdown list by ADE case* |
| ADE Symptom 1 | *Preloaded from dropdown list by ADE case* |
| ADE Symptom 2 | *Preloaded from dropdown list by ADE case* |
| Other ADE Symptom | *Preloaded from dropdown list by ADE case* |
| ADE Diagnosis | *Preloaded from dropdown list by ADE case* |
| Form Status | |
| Complete? | *Complete/Unverified/Incomplete* |

**MedDRA** **[RA1]** (not visible to RA2)

| Record ID | Automatically assigned by REDCap (numbered 1-573) |
| --- | --- |
| Please describe the symptom(s) and/or diagnosis in the event below using MedDRA (US) terminology.  [*ADE ID*] involved the following drug(s): [*ADE Drug 1*] [*ADE Drug 2*] [*ADE Drug 3*] [*Other ADE Drug 1*] [*Other ADE Drug 2*]  This ADE resulted in a diagnosis of [ADE Diagnosis] and the following symptom(s): [*ADE Symptom 1*] [*ADE Symptom 2*] [*Other ADE Symptom*] | |
| Is there sufficient ADE case information available to properly classify all symptoms/diagnoses for this event? | *Yes/No* |
| MedDRA (US) 1 | *Choose from MedDRA_US dropdown list* |
| Please indicate your degree of comfort with the above rating: | *Very uncomfortable/ Uncomfortable/ Neutral/ Comfortable/ Very comfortable* |
| MedDRA (US) 2 | *Choose from MedDRA_US dropdown list* |
| Please indicate your degree of comfort with the above rating: | *Very uncomfortable/ Uncomfortable/ Neutral/ Comfortable/ Very comfortable* |
| MedDRA (US) 3 | *Choose from MedDRA_US dropdown list* |
| Please indicate your degree of comfort with the above rating: | *Very uncomfortable/ Uncomfortable/ Neutral/ Comfortable/ Very comfortable* |
| MedDRA (US) 4 | *Choose from MedDRA_US dropdown list* |
| Please indicate your degree of comfort with the above rating: | *Very uncomfortable/ Uncomfortable/ Neutral/ Comfortable/ Very comfortable* |
| Are the MedDRA terms entered above sufficient enough to accurately describe this case? | *Yes/No/Unclear* |
| Notes/Comments |  |
| Form Status | |
| Complete? | *Complete/Unverified/Incomplete* |

**SNOMED (Adverse Reactions)** **[RA1]** (not visible to RA2)

| Record ID | Automatically assigned by REDCap (numbered 1-573) |
| --- | --- |
| Please describe the symptom(s) and/or diagnosis in the event below using SNOMED (ADR) terminology.  [*ADE ID*] involved the following drug(s): [*ADE Drug 1*] [*ADE Drug 2*] [*ADE Drug 3*] [*Other ADE Drug 1*] [*Other ADE Drug 2*]  This ADE resulted in a diagnosis of [ADE Diagnosis] and the following symptom(s): [*ADE Symptom 1*] [*ADE Symptom 2*] [*Other ADE Symptom*] | |
| Is there sufficient ADE case information available to properly classify all symptoms/diagnoses for this event? | *Yes/No* |
| SNOMED (ADR) 1 | *Choose from SNOMED_ADR dropdown list* |
| Please indicate your degree of comfort with the above rating: | *Very uncomfortable/ Uncomfortable/ Neutral/ Comfortable/ Very comfortable* |
| SNOMED (ADR) 2 | *Choose from SNOMED_ADR dropdown list* |
| Please indicate your degree of comfort with the above rating: | *Very uncomfortable/ Uncomfortable/ Neutral/ Comfortable/ Very comfortable* |
| SNOMED (ADR) 3 | *Choose from SNOMED_ADR dropdown list* |
| Please indicate your degree of comfort with the above rating: | *Very uncomfortable/ Uncomfortable/ Neutral/ Comfortable/ Very comfortable* |
| SNOMED (ADR) 4 | *Choose from SNOMED_ADR dropdown list* |
| Please indicate your degree of comfort with the above rating: | *Very uncomfortable/ Uncomfortable/ Neutral/ Comfortable/ Very comfortable* |
| Are the SNOMED ADR terms entered above sufficient enough to accurately describe this case? | *Yes/No/Unclear* |
| Notes/Comments |  |
| Form Status | |
| Complete? | *Complete/Unverified/Incomplete* |

**SNOMED (Health Concerns)** **[RA1]** (not visible to RA2)

| Record ID | Automatically assigned by REDCap (numbered 1-573) |
| --- | --- |
| Please describe the symptom(s) and/or diagnosis in the event below using SNOMED (HC) terminology.  [*ADE ID*] involved the following drug(s): [*ADE Drug 1*] [*ADE Drug 2*] [*ADE Drug 3*] [*Other ADE Drug 1*] [*Other ADE Drug 2*]  This ADE resulted in a diagnosis of [ADE Diagnosis] and the following symptom(s): [*ADE Symptom 1*] [*ADE Symptom 2*] [*Other ADE Symptom*] | |
| Is there sufficient ADE case information available to properly classify all symptoms/diagnoses for this event? | *Yes/No* |
| SNOMED (HC)1 | *Choose from SNOMED_HC dropdown list* |
| Please indicate your degree of comfort with the above rating: | *Very uncomfortable/ Uncomfortable/ Neutral/ Comfortable/ Very comfortable* |
| SNOMED (HC)2 | *Choose from SNOMED_HC dropdown list* |
| Please indicate your degree of comfort with the above rating: | *Very uncomfortable/ Uncomfortable/ Neutral/ Comfortable/ Very comfortable* |
| SNOMED (HC) 3 | *Choose from SNOMED_HC* *dropdown list* |
| Please indicate your degree of comfort with the above rating: | *Very uncomfortable/ Uncomfortable/ Neutral/ Comfortable/ Very comfortable* |
| SNOMED (HC) 4 | *Choose from SNOMED_HC* *dropdown list* |
| Please indicate your degree of comfort with the above rating: | *Very uncomfortable/ Uncomfortable/ Neutral/ Comfortable/ Very comfortable* |
| Are the SNOMED (HC) terms entered above sufficient enough to accurately describe this case? | *Yes/No/Unclear* |
| Notes/Comments |  |
| Form Status | |
| Complete? | *Complete/Unverified/Incomplete* |

**ICD-11** **[RA1]** (not visible to RA2)

| Record ID | Automatically assigned by REDCap (numbered 1-573) |
| --- | --- |
| Please describe the symptom(s) and/or diagnosis in the event below using ICD terminology.  [*ADE ID*] involved the following drug(s): [*ADE Drug 1*] [*ADE Drug 2*] [*ADE Drug 3*] [*Other ADE Drug 1*] [*Other ADE Drug 2*]  This ADE resulted in a diagnosis of [ADE Diagnosis] and the following symptom(s): [*ADE Symptom 1*] [*ADE Symptom 2*] [*Other ADE Symptom*] | |
| Is there sufficient ADE case information available to properly classify all symptoms/diagnoses for this event? | *Yes/No* |
| ICD-11 (1) | *Choose from ICD11 dropdown list* |
| Please indicate your degree of comfort with the above rating: | *Very uncomfortable/ Uncomfortable/ Neutral/ Comfortable/ Very comfortable* |
| ICD-11 (2) | *Choose from ICD11 dropdown list* |
| Please indicate your degree of comfort with the above rating: | *Very uncomfortable/ Uncomfortable/ Neutral/ Comfortable/ Very comfortable* |
| ICD-11 (3) | *Choose from ICD11* *dropdown list* |
| Please indicate your degree of comfort with the above rating: | *Very uncomfortable/ Uncomfortable/ Neutral/ Comfortable/ Very comfortable* |
| ICD-11 (4) | *Choose from ICD11* *dropdown list* |
| Please indicate your degree of comfort with the above rating: | *Very uncomfortable/ Uncomfortable/ Neutral/ Comfortable/ Very comfortable* |
| Are the ICD-11 terms entered above sufficient enough to accurately describe this case? | *Yes/No/Unclear* |
| Notes/Comments |  |
| Form Status | |
| Complete? | *Complete/Unverified/Incomplete* |

**MedDRA** **[RA2]** (not visible to RA1)

| Record ID | Automatically assigned by REDCap (numbered 1-573) |
| --- | --- |
| Please describe the symptom(s) and/or diagnosis in the event below using MedDRA (US) terminology.  [*ADE ID*] involved the following drug(s): [*ADE Drug 1*] [*ADE Drug 2*] [*ADE Drug 3*] [*Other ADE Drug 1*] [*Other ADE Drug 2*]  This ADE resulted in a diagnosis of [ADE Diagnosis] and the following symptom(s): [*ADE Symptom 1*] [*ADE Symptom 2*] [*Other ADE Symptom*] | |
| Is there sufficient ADE case information available to properly classify all symptoms/diagnoses for this event? | *Yes/No* |
| MedDRA (US) 1 | *Choose from MedDRA_US dropdown list* |
| Please indicate your degree of comfort with the above rating: | *Very uncomfortable/ Uncomfortable/ Neutral/ Comfortable/ Very comfortable* |
| MedDRA (US) 2 | *Choose from MedDRA_US dropdown list* |
| Please indicate your degree of comfort with the above rating: | *Very uncomfortable/ Uncomfortable/ Neutral/ Comfortable/ Very comfortable* |
| MedDRA (US) 3 | *Choose from MedDRA_US dropdown list* |
| Please indicate your degree of comfort with the above rating: | *Very uncomfortable/ Uncomfortable/ Neutral/ Comfortable/ Very comfortable* |
| MedDRA (US) 4 | *Choose from MedDRA_US dropdown list* |
| Please indicate your degree of comfort with the above rating: | *Very uncomfortable/ Uncomfortable/ Neutral/ Comfortable/ Very comfortable* |
| Are the MedDRA terms entered above sufficient enough to accurately describe this case? | *Yes/No/Unclear* |
| Notes/Comments |  |
| Form Status | |
| Complete? | *Complete/Unverified/Incomplete* |

**SNOMED (Adverse Reactions)** **[RA2]** (not visible to RA1)

| Record ID | Automatically assigned by REDCap (numbered 1-573) |
| --- | --- |
| Please describe the symptom(s) and/or diagnosis in the event below using SNOMED (ADR) terminology.  [*ADE ID*] involved the following drug(s): [*ADE Drug 1*] [*ADE Drug 2*] [*ADE Drug 3*] [*Other ADE Drug 1*] [*Other ADE Drug 2*]  This ADE resulted in a diagnosis of [ADE Diagnosis] and the following symptom(s): [*ADE Symptom 1*] [*ADE Symptom 2*] [*Other ADE Symptom*] | |
| Is there sufficient ADE case information available to properly classify all symptoms/diagnoses for this event? | *Yes/No* |
| SNOMED (ADR) 1 | *Choose from SNOMED_ADR dropdown list* |
| Please indicate your degree of comfort with the above rating: | *Very uncomfortable/ Uncomfortable/ Neutral/ Comfortable/ Very comfortable* |
| SNOMED (ADR) 2 | *Choose from SNOMED_ADR dropdown list* |
| Please indicate your degree of comfort with the above rating: | *Very uncomfortable/ Uncomfortable/ Neutral/ Comfortable/ Very comfortable* |
| SNOMED (ADR) 3 | *Choose from SNOMED_ADR dropdown list* |
| Please indicate your degree of comfort with the above rating: | *Very uncomfortable/ Uncomfortable/ Neutral/ Comfortable/ Very comfortable* |
| SNOMED (ADR) 4 | *Choose from SNOMED_ADR dropdown list* |
| Please indicate your degree of comfort with the above rating: | *Very uncomfortable/ Uncomfortable/ Neutral/ Comfortable/ Very comfortable* |
| Are the SNOMED ADR terms entered above sufficient enough to accurately describe this case? | *Yes/No/Unclear* |
| Notes/Comments |  |
| Form Status | |
| Complete? | *Complete/Unverified/Incomplete* |

**SNOMED (Health Concerns)** **[RA2]** (not visible to RA1)

| Record ID | Automatically assigned by REDCap (numbered 1-573) |
| --- | --- |
| Please describe the symptom(s) and/or diagnosis in the event below using SNOMED (HC) terminology.  [*ADE ID*] involved the following drug(s): [*ADE Drug 1*] [*ADE Drug 2*] [*ADE Drug 3*] [*Other ADE Drug 1*] [*Other ADE Drug 2*]  This ADE resulted in a diagnosis of [ADE Diagnosis] and the following symptom(s): [*ADE Symptom 1*] [*ADE Symptom 2*] [*Other ADE Symptom*] | |
| Is there sufficient ADE case information available to properly classify all symptoms/diagnoses for this event? | *Yes/No* |
| SNOMED (HC)1 | *Choose from SNOMED_HC dropdown list* |
| Please indicate your degree of comfort with the above rating: | *Very uncomfortable/ Uncomfortable/ Neutral/ Comfortable/ Very comfortable* |
| SNOMED (HC)2 | *Choose from SNOMED_HC dropdown list* |
| Please indicate your degree of comfort with the above rating: | *Very uncomfortable/ Uncomfortable/ Neutral/ Comfortable/ Very comfortable* |
| SNOMED (HC) 3 | *Choose from SNOMED_HC* *dropdown list* |
| Please indicate your degree of comfort with the above rating: | *Very uncomfortable/ Uncomfortable/ Neutral/ Comfortable/ Very comfortable* |
| SNOMED (HC) 4 | *Choose from SNOMED_HC* *dropdown list* |
| Please indicate your degree of comfort with the above rating: | *Very uncomfortable/ Uncomfortable/ Neutral/ Comfortable/ Very comfortable* |
| Are the SNOMED (HC) terms entered above sufficient enough to accurately describe this case? | *Yes/No/Unclear* |
| Notes/Comments |  |
| Form Status | |
| Complete? | *Complete/Unverified/Incomplete* |

**ICD-11** **[RA2]** (not visible to RA1)

| Record ID | Automatically assigned by REDCap (numbered 1-573) |
| --- | --- |
| Please describe the symptom(s) and/or diagnosis in the event below using ICD terminology.  [*ADE ID*] involved the following drug(s): [*ADE Drug 1*] [*ADE Drug 2*] [*ADE Drug 3*] [*Other ADE Drug 1*] [*Other ADE Drug 2*]  This ADE resulted in a diagnosis of [ADE Diagnosis] and the following symptom(s): [*ADE Symptom 1*] [*ADE Symptom 2*] [*Other ADE Symptom*] | |
| Is there sufficient ADE case information available to properly classify all symptoms/diagnoses for this event? | *Yes/No* |
| ICD-11 (1) | *Choose from ICD11 dropdown list* |
| Please indicate your degree of comfort with the above rating: | *Very uncomfortable/ Uncomfortable/ Neutral/ Comfortable/ Very comfortable* |
| ICD-11 (2) | *Choose from ICD11 dropdown list* |
| Please indicate your degree of comfort with the above rating: | *Very uncomfortable/ Uncomfortable/ Neutral/ Comfortable/ Very comfortable* |
| ICD-11 (3) | *Choose from ICD11* *dropdown list* |
| Please indicate your degree of comfort with the above rating: | *Very uncomfortable/ Uncomfortable/ Neutral/ Comfortable/ Very comfortable* |
| ICD-11 (4) | *Choose from ICD11* *dropdown list* |
| Please indicate your degree of comfort with the above rating: | *Very uncomfortable/ Uncomfortable/ Neutral/ Comfortable/ Very comfortable* |
| Are the ICD-11 terms entered above sufficient enough to accurately describe this case? | *Yes/No/Unclear* |
| Notes/Comments |  |
| Form Status | |
| Complete? | *Complete/Unverified/Incomplete* |
