# Supplementary material for: The Utility of Different Data Standards to Document Adverse Drug Event Symptoms and Diagnoses: Mixed Methods Study
Source: J Med Internet Res. 2021 Dec 10;23(12):e27188. doi: 10.2196/27188 (PMC8709916; doi:10.2196/27188)
Supplement: Multimedia Appendix 3 [file jmir_v23i12e27188_app3.docx]

## Appendix 3. Consensus data collection form.

**Consensus – MedDRA** (only visible after both RAs have completed the earlier forms and only if there is discordance between their answers for these questions)

| Record ID | Automatically assigned by REDCap (numbered 1-573) |
| --- | --- |
| [*ADE ID*] involved the following drug(s): [*ADE Drug 1*] [*ADE Drug 2*] [*ADE Drug 3*] [*Other ADE Drug 1*] [*Other ADE Drug 2*]  This ADE resulted in a diagnosis of [ADE Diagnosis] and the following symptom(s): [*ADE Symptom 1*] [*ADE Symptom 2*] [*Other ADE Symptom*]  1) Is there sufficient ADE case information available to properly classify all symptoms and/or diagnoses for this event using MedDRA?  RA1 said: [*meddra_info_RA1*]  RA2 said: [*meddra_info_RA2*]  2) Are the MedDRA terms provided sufficient enough to accurately describe this case?  RA1 said: [*meddra_sufficient_RA1*] = [*meddra1_RA1*] [*meddra2_RA1*] [*meddra3_RA1*] [*meddra4_RA1*]  RA2 said: [*meddra_sufficient_RA2*] = [*meddra1_RA2*] [*meddra2_RA2*] [*meddra3_RA2*] [*meddra4_RA2*] | |
| Consensus Rating: Is there sufficient ADE case information available to properly classify all symptoms/diagnoses for this event? | *Yes/No* |
| Consensus Rating: Are the MedDRA terms entered above sufficient enough to accurately describe this case? | *Yes/No* |
| Notes/Comments |  |
| Form Status | |
| Complete? | *Complete/Unverified/Incomplete* |

**Consensus – SNOMED ADR** (only visible after both RAs have completed the earlier forms and only if there is discordance between their answers for these questions)

| Record ID | Automatically assigned by REDCap (numbered 1-573) |
| --- | --- |
| [*ADE ID*] involved the following drug(s): [*ADE Drug 1*] [*ADE Drug 2*] [*ADE Drug 3*] [*Other ADE Drug 1*] [*Other ADE Drug 2*]  This ADE resulted in a diagnosis of [ADE Diagnosis] and the following symptom(s): [*ADE Symptom 1*] [*ADE Symptom 2*] [*Other ADE Symptom*]  1) Is there sufficient ADE case information available to properly classify all symptoms and/or diagnoses for this event using SNOMED (ADR)?  RA1 said: [*snomed_adr_info_RA1*]  RA2 said: [*snomed_adr _info_RA2*]  2) Are the SNOMED (ADR) terms provided sufficient enough to accurately describe this case?  RA1 said: [*snomed_adr_sufficient_RA1*] = [*snomed_adr1_RA1*] [*snomed_adr2_RA1*] [*snomed_adr3_RA1*] [*snomed_adr4_RA1*]  RA2 said: [*snomed_adr_sufficient_RA2*] = [*snomed_adr1_RA2*] [*snomed_adr2_RA2*] [*snomed_adr3_RA2*] [*snomed_adr4_RA2*] | |
| Consensus Rating: Is there sufficient ADE case information available to properly classify all symptoms/diagnoses for this event? | *Yes/No* |
| Consensus Rating: Are the SNOMED (ADR) terms entered above sufficient enough to accurately describe this case? | *Yes/No* |
| Notes/Comments |  |
| Form Status | |
| Complete? | *Complete/Unverified/Incomplete* |

**Consensus – SNOMED HC** (only visible after both RAs have completed the earlier forms and only if there is discordance between their answers for one or both of these questions)

| Record ID | Automatically assigned by REDCap (numbered 1-573) |
| --- | --- |
| [*ADE ID*] involved the following drug(s): [*ADE Drug 1*] [*ADE Drug 2*] [*ADE Drug 3*] [*Other ADE Drug 1*] [*Other ADE Drug 2*]  This ADE resulted in a diagnosis of [ADE Diagnosis] and the following symptom(s): [*ADE Symptom 1*] [*ADE Symptom 2*] [*Other ADE Symptom*]  1) Is there sufficient ADE case information available to properly classify all symptoms and/or diagnoses for this event using SNOMED (HC)?  RA1 said: [*snomed_hc_info_RA1*]  RA2 said: [*snomed_hc _info_RA2*]  2) Are the SNOMED (HC) terms provided sufficient enough to accurately describe this case?  RA1 said: [*snomed_hc_sufficient_RA1*] = [*snomed_hc1_RA1*] [*snomed_hc2_RA1*] [*snomed_hc3_RA1*] [*snomed_hc4_RA1*]  RA2 said: [*snomed_hc _sufficient_RA2*] = [*snomed_hc1_RA2*] [*snomed_hc2_RA2*] [*snomed_hc3_RA2*] [*snomed_hc4_RA2*] | |
| Consensus Rating: Is there sufficient ADE case information available to properly classify all symptoms/diagnoses for this event? | *Yes/No* |
| Consensus Rating: Are the SNOMED (HC) terms entered above sufficient enough to accurately describe this case? | *Yes/No* |
| Notes/Comments |  |
| Form Status | |
| Complete? | *Complete/Unverified/Incomplete* |

**Consensus – ICD-11** (only visible after both RAs have completed the earlier forms and only if there is discordance between their answers for one or both of these questions)

| Record ID | Automatically assigned by REDCap (numbered 1-573) |
| --- | --- |
| [*ADE ID*] involved the following drug(s): [*ADE Drug 1*] [*ADE Drug 2*] [*ADE Drug 3*] [*Other ADE Drug 1*] [*Other ADE Drug 2*]  This ADE resulted in a diagnosis of [ADE Diagnosis] and the following symptom(s): [*ADE Symptom 1*] [*ADE Symptom 2*] [*Other ADE Symptom*]  1) Is there sufficient ADE case information available to properly classify all symptoms and/or diagnoses for this event using ICD-11?  RA1 said: [*icd_info_RA1*]  RA2 said: [*icd_info_RA2*]  2) Are the ICD-11 terms provided sufficient enough to accurately describe this case?  RA1 said: [*icd_sufficient_RA1*] = [*icd1_RA1*] [*icd2_RA1*] [*icd3_RA1*] [*icd4_RA1*]  RA2 said: [*icd_sufficient_RA2*] = [*icd1_RA2*] [*icd2_RA2*] [*icd3_RA2*] [*icd4_RA2*] | |
| Consensus Rating: Is there sufficient ADE case information available to properly classify all symptoms/diagnoses for this event? | *Yes/No* |
| Consensus Rating: Are the ICD-11 terms entered above sufficient enough to accurately describe this case? | *Yes/No* |
| Notes/Comments |  |
| Form Status | |
| Complete? | *Complete/Unverified/Incomplete* |
