# Supplementary material for: The Utility of Different Data Standards to Document Adverse Drug Event Symptoms and Diagnoses: Mixed Methods Study
Source: J Med Internet Res. 2021 Dec 10;23(12):e27188. doi: 10.2196/27188 (PMC8709916; doi:10.2196/27188)
Supplement: Multimedia Appendix 4 [file jmir_v23i12e27188_app4.docx]

**Appendix 4. Adverse Drug Events with no Matching Terms.**

**SNOMED Health Concern & Diagnosis (SNOMED HC)**

| **ADE Type** | **ADE Drug(s)** | **ADE Symptoms/**  **Diagnoses** | **SNOMED_HC RA1** | **SNOMED_HC RA2** |
| --- | --- | --- | --- | --- |
| Low Dose | WARFARIN | INR subtherapeutic/  Lab abnormality | Coagulation/ bleeding tests abnormal | Dizziness |
| Non-Adherence | QUETIAPINE  RISPERIDONE  VALPROIC ACID | Abnormal behaviour; Suicidal/  Psychosis | Psychotic disorder; Injury due to suicide attempt; Delusions; Auditory hallucinations | Schizophrenia; Affective psychosis |
| Adverse Drug Reaction | TINZAPARIN  WARFARIN | Hematuria | Frank hematuria | Coagulation/ bleeding tests abnormal; Blood in urine |
| Adverse Drug Reaction | MEPERIDINE | Rash/  Allergic reaction | Lipedema; Allergic disorder of skin | Swelling/lump finding; Edema |
| Needs Additional Drug/Untreated Indication | ACETAMINOPHEN  CODEINE  FLUOXETINE  CAFFEINE  OXYCODONE | Medication required | Drug withdrawal | Symptoms of depression |
| High Dose | LITHIUM | Abnormal lab value | No match | Hallucinations |
| Adverse Drug Reaction | CANDESARTAN | Congested ears; Throat scratchy; Swollen external neck | Sensation of blocked ears; Pain in throat; Feeling of lump in throat; Lymphadenopathy | Congestion of nasal sinus; Choking sensation |
| High Dose | CLOBAZAM | Weakness | No match | Ataxia |
| Adverse Drug Reaction | AMOXICILLIN | Hives; Rash/  Allergic reaction | Allergic disorder of skin | Idiopathic urticaria |
| Adverse Drug Reaction | ACETYLSALICYLIC ACID | Bleeding; Oral bleeding/  Bleed (not GI) | No match | Coagulation/ bleeding tests abnormal |
| Adverse Drug Reaction | RIVAROXABAN | Hematuria/  Bleed (not GI) | Frank hematuria | Blood in urine |
| Ineffective Drug | FLUCINOLONE | Rash | No match | Viral exanthem |
| Ineffective Drug | ZINC PRODUCTS  HYDROCORTISONE  FIBER | Blood in stool | Hematochezia | Occult blood in stools; Melena |
| Non-Adherence | TRAZODONE  SERTRALINE  QUETIAPINE  METHOTRIMEPRA-ZINE | Suicidal | Fatigue | Generalized anxiety disorder; Bipolar disorder |
| Non-Adherence | RISPERIDONE | Refusal to take meds or leave home/  Schizophrenia | Noncompliance with treatment | Schizophrenia |
| Adverse Drug Reaction | HYDROMORPHONE | Weird dreams | Dream anxiety disorder | No match |
| Adverse Drug Reaction | CIPROFLOXACIN | Rash; Pruritus/  Allergic reaction | Allergic disorder of skin | No match |
| High Dose | METOPROLOL | Bradycardia/  Falls | Drug-induced bradycardia; Syncope | Bradycardia; Falls |
| Non-Adherence | RISPERIDONE | Suicidal/  Psychosis | Psychotic disorder | Schizophrenia |
| Non-Adherence | OLANZAPINE  CLONAZEPAM  PROCYCLIDINE | Paranoia/  Psychosis | Psychotic disorder | Reactive psychoses; Paranoid schizophrenia |
| Drug Interaction | DIPHENHYDRA-MINE  HYDROMORPHONE  MORPHINE | Urinary retention | Retention of urine | Urinary incontinence |
| Non-Adherence | QUETIAPINE | Paranoia; Agitation/  Psychosis | Delusions; Psychotic disorder | Affective psychosis; Feeling agitated; Paranoid schizophrenia |
| Needs Additional Drug/Untreated Indication | NORTRIPTYLINE  PREGABALIN | Pain; Med request/  Neuropathic pain | Neuropathic pain | Pain |
| Adverse Drug Reaction | GENTAMICIN  CIPROFLOXACIN | Thrush | Candidiasis of mouth | No match |
| Non-Adherence | QUETIAPINE  LITHIUM | Suicidal; Depression/  Bipolar disease | Injury due to suicide attempt; Bipolar affective disorder, currently depressed, severe, with psychosis | Symptoms of depression; Bipolar disorder |
| Adverse Drug Reaction | DEXAMETHASONE | Pedal edema | Peripheral edema | Edema of foot |
| Drug Interaction | SIROLIMUS  TACROLIMUS  CLARITHROMYCIN | Abnormal lab values; Vomiting | Nausea and vomiting | Vomiting |
| Adverse Drug Reaction | DOXYCYCLINE | Odynophagia | Swallowing painful | Dysphagia |
| Ineffective Drug | CLINDAMYCIN | Purulence; Foul smell/  Infection other | Postoperative infection | Post-traumatic wound infection |
| Needs Additional Drug/Untreated Indication | AMLODIPINE | Hypertension; Troponin increase/  Hypertension | Finding of increased blood pressure | Primary hypertension |
| Low Dose | FUROSEMIDE | Edema; Swelling of feet and forearms/  Edema | Peripheral edema | Edema of foot |
| Non-Adherence | LITHIUM  FLUPENTIXOL | Paranoia; Abnormal behaviour/  Psychosis | Delusions; Psychotic disorder | Paranoid schizophrenia; Mixed bipolar affective disorder, severe, with psychosis |
| Needs Additional Drug/Untreated Indication | MIRTAZAPINE | Depression | Symptoms of depression | Depressive disorder |
| Adverse Drug Reaction | RIVAROXABAN | Vaginal bleed | No match | Ovulation bleeding |
| Needs Additional Drug/Untreated Indication | ACETYLSALICYLIC ACID | Word finding difficulties | Anomic aphasia | Difficulty using verbal communication; Transient ischemic attack |
| Non-Adherence | RISPERIDONE | Psychosis; Abnormal behaviour/  Psychosis | Psychotic disorder | Affective psychosis |
| High Dose | BROMOCRIPTINE | EPS | Medication-induced movement disorder | No match |
| Adverse Drug Reaction | RIVASTIGMINE | Nausea; Diarrhea; Collapse/  Weakness | Nausea and vomiting; Diarrhea | Asthenia; Nausea |
| Low Dose | MEDROXY-PROGESTERONE | Vaginal bleed/  Bleed (not GI) | No match | Endometriosis |
| Non-Adherence | QUETIAPINE  RISPERIDONE  VALPROIC ACID | Abnormal behaviour; Suicidal/  Psychosis | Psychotic disorder; Auditory hallucinations | Injury due to suicide attempt; Affective psychosis |
| Non-Adherence | HALOPERIDOL | Rigid; Stiff | Spasticity | Muscle rigidity |
| Needs Additional Drug/Untreated Indication | WARFARIN | Leg pain/  Deep vein thrombosis | Thrombophlebitis of deep veins of lower extremity | Deep vein thrombosis |
| Needs Additional Drug/Untreated Indication | ACETYLSALICYLIC ACID | Slurred speech | Dysarthria | Slurred speech |
| Drug Interaction | VENLAFAXINE  PRIMIDONE  MIRTAZAPINE  METOCLOPRAMIDE | Delirium; Tremor; Weakness; Diarrhea | Serotonin syndrome | Delirium; Tremor; Diarrhea |

**SNOMED Adverse Reaction (SNOMED ADR)**

| **ADE Type** | **ADE Drug(s)** | **ADE Symptoms/**  **Diagnoses** | **SNOMED_ADR RA1** | **SNOMED_ADR RA2** |
| --- | --- | --- | --- | --- |
| Low Dose | WARFARIN | Abnormal lab values/  Lab abnormality | Coagulation/ bleeding tests abnormal | Clotting time above reference range |
| Low Dose | WARFARIN | INR subtherapeutic/  Lab abnormality | Coagulation/ bleeding tests abnormal | Dizziness |
| Adverse Drug Reaction | TINZAPARIN  WARFARIN | Hematuria | Frank hematuria | Bleeding; Coagulation/ bleeding tests abnormal |
| Adverse Drug Reaction | EPINEPHRINE | Headache | No Match | Frontal headache |
| Needs Additional Drug/Untreated Indication | ACETAMINOPHEN  OXYCODONE  CODEINE  CAFFEINE  FLUOXETINE | Medication required | No Match | Moderate depression |
| Non-Adherence | ERGOTAMINE  CAFFEINE | Headache/  Migraine | Photosensitivity | Frontal headache; Migraine without aura |
| High Dose | LITHIUM | Abnormal lab values | No Match | Hallucination |
| Adverse Drug Reaction | PAPVERINE  PROSTAGLANDINS | Priapism | No Match | Pain |
| Needs Additional Drug/Untreated Indication | PROGESTERONE | Miscarriage | Generalized abdominal pain | Complete abortion |
| Adverse Drug Reaction | GEMCITABINE  CISPLATIN  DEXAMETHASONE | Pancytopenia | Chemotherapy-induced neutropenia; Leukopenia; Thrombocyto-penic disorder; Anemia due to chemotherapy | Aplastic anemia |
| Adverse Drug Reaction | RIVAROXABAN | Fall/  Subarachnoid hemorrhage | Bleeding | No match |
| Adverse Drug Reaction | DESMOPRESSIN | Hyponatremia; Abdominal pain; Urinary retention/  Lab abnormality | Hyponatremia; Blood chemistry Abnormal; Nausea; Generalized abdominal pain | Hypo-osmolality and/or hyponatremia; Abdominal discomfort |
| Needs Additional Drug/Untreated Indication | FENTANYL | Withdrawal | No Match | Pain |
| Adverse Drug Reaction | NSAIDS | Medication overuse headache/  Headache | No Match | Migraine without aura; Muscle weakness |
| Adverse Drug Reaction | ACETAYLSALICYLIC ACID | Bleeding; Oral bleeding/  Bleed (not GI) | Bleeding | Bleeding gums |
| High Dose | WARFARIN | Abnormal lab values; Elevated INR/  Lab abnormality | Coagulation/ bleeding tests abnormal | International Normalized Ratio raised |
| Non-Adherence | OLANZAPINE | Abnormal lab values; Abnormal behaviour/  Mania | Problem behaviour | Psychotic disorder |
| Ineffective Drug | ZINC PRODUCTS  HYDROCORTISONE  FIBER | Blood in stool | Hematochezia | Occult blood in stools |
| Needs Additional Drug/Untreated Indication | WARFARIN | Headache | No Match | Frontal headache |
| Non-Adherence | LITHIUM  QUETIAPINE | Abnormal behaviour/  Mania | Problem behaviour | Hyperactive behaviour |
| Non-Adherence | RISPERIDONE | Refusal to take meds or leave home/  Schizophrenia | No Match | Psychotic disorder |
| Non-Adherence | HALOPERIDOL | Rigid; Stiff | No Match | Spasticity |
| Adverse Drug Reaction | ZOPICLONE | Metallic taste in mouth | Disorder of taste | No match |
| Low Dose | VALPROIC ACID | Abnormal behaviour/  Bipolar disease | Problem behaviour | Hyperactive behaviour; Psychotic disorder |
| High Dose | MORPHINE | Fall/  Fracture | Drowsy | No match |
| Adverse Drug Reaction | PREDNISONE | Fall; Weakness | Muscle weakness | Asthenia |
| Non-Adherence | CIPROFLOXACIN | Hematuria/  Urinary tract infection | Frank hematuria; Urinary symptoms | Lower urinary tract symptoms; Blood in urine |
| Adverse Drug Reaction | ZOPICLONE | Weakness; Dizziness/  Falls | Muscle weakness; Syncope and collapse | Asthenia; Dizziness |
| Drug Interaction | LORAZEPAM  OXAZEPAM | Syncope; Falls/  Falls | Syncope and collapse | Syncope |
| Needs Additional Drug/Untreated Indication | WARFARIN | Confusion/  Stroke | Delusions | No match |
| High Dose | WARFARIN | INR subtherapeutic; Bleeding/  Lab abnormality | Coagulation/ bleeding tests abnormal; Anemia | International Normalized Ratio raised; Bleeding |
| Adverse Drug Reaction | GLYBURIDE | Hypoglycemia | Drug-induced hypoglycemia | Hypoglycemia |
| Needs Additional Drug/Untreated Indication | NIFEDIPINE | Hypertension; Chest pain/  Hypertension | Abnormal blood pressure; Atypical chest pain | Systolic hypertension; Chest discomfort |
| Non-Adherence | SIMVASTATIN  RAMIPRIL | Leg pain/  Ischemia | Pain in lower limb | Pain |
| Adverse Drug Reaction | GENTAMICIN  CIPROFLOXACIN | Thrush | Candidiasis of mouth | No match |
| Needs Additional Drug/Untreated Indication | WARFARIN | Slurred speech; Facial asymmetry/  Stroke | Dysarthria | Disturbance in speech |
| Adverse Drug Reaction | DEXAMETHASONE | Pedal edema | Ankle edema | Peripheral edema |
| Adverse Drug Reaction | RITUXIMAB  CARBOPLATIN  IFOSFAMIDE  ETOPOSIDE | Weakness; Pancytopenia | Muscle weakness; Coagulation/ bleeding tests abnormal; Anemia; Leukopenia | Asthenia |
| Adverse Drug Reaction | WARFARIN | Headache; Confusion/  Subdural hematoma | No match | Frontal headache |
| Drug Interaction | SIROLIMUS  TACROLIMUS  CLARITHROMYCIN | Abnormal lab values; Vomiting | Drug level above therapeutic; Nausea; Vomiting | Vomiting |
| Non-Adherence | WARFARIN  SIMVASTATIN | Altered level of consciousness/  Stroke | Unconscious | Clouded consciousness |
| Needs Additional Drug/Untreated Indication | WARFARIN | Facial droop; Unable to verbalize/  Stroke | Aphasia | Numbness of face |
| Adverse Drug Reaction | DOXYCYCLINE | Odynophagia | Swallowing painful | Dysphagia |
| Low Dose | WARFARIN | INR subtherapeutic/  Pulmonary embolism | Coagulation/ bleeding tests abnormal | No match |
| Non-Adherence | GLICLAZIDE | Hypoglycemia; Fall/  Hypoglycemia | Drug-induced hypoglycemia | Hypoglycemia |
| Low Dose | FUROSEMIDE | Edema; Swelling of feet and forearms/  Edema | Peripheral edema | Edema of foot |
| Needs Additional Drug/Untreated Indication | WARFARIN | Slurred speech; Weakness | Muscle weakness; dysarthria | Disturbance in speech; Asthenia |
| Adverse Drug Reaction | ACETYLSALICYLIC ACID | Headache/  Subarachnoid hemorrhage | No Match | Bleeding; Migraine without aura |
| Adverse Drug Reaction | ALLOPURINOL | Rash; Altered level of consciousness/  Allergic reaction | Rash-eruption of skin | Allergic urticaria; Clouded consciousness |
| Needs Additional Drug/Untreated Indication | ACETYLSALICYLIC ACID | Word finding difficulties | Aphasia | Difficulty speaking |
| High Dose | BROMOCRIPTINE | EPS | Abnormal involuntary movement | No match |
| High Dose | INSULIN (HUMAN)  GLICLAZIDE | Confusion/  Hypoglycemia | Drug-induced hypoglycemia | Hypoglycemia |
| Adverse Drug Reaction | DILTIAZEM  TIMOLOL | Other | No Match | Bradycardia |
| Non-Adherence | INSULIN (HUMAN) | Fall; Hypoglycemia/  Hypoglycemia | Drug-induced hypoglycemia | Hypoglycemia |
| Needs Additional Drug/Untreated Indication | WARFARIN | Chest pain/  Pulmonary embolism | No Match | Chest discomfort |
| Adverse Drug Reaction | GLYBURIDE | Confusion; Hypoglycemia/  Hypoglycemia | Drug-induced hypoglycemia | Hypoglycemia |
| Drug Interaction | WARFARIN  PRASUGREL  ACETYLSALICYLIC ACID | Rectal bleed; Blood in stool; INR supratherapeutic | Hematochezia; Coagulation/ bleeding tests abnormal | Melena; International Normalized Ratio raised |
| Needs Additional Drug/Untreated Indication | WARFARIN | Confusion; Vision loss/  Stroke | No match | Hazy vision |
| Adverse Drug Reaction | RIVASTIGMINE | Nausea; Diarrhea; Collapse/  Weakness | Nausea and vomiting; Diarrhea | Asthenia; Nausea |
| High Dose | WARFARIN | Dizziness; Abnormal lab values/  Falls | Dizziness and giddiness | Dizziness |
| Adverse Drug Reaction | NITROFURANTOIN | Rash; Lips swollen; Itchy throat/  Allergic reaction | Hypersensitivity reaction; Rash-eruption of skin; Angio-odema of lips | Allergic urticaria; Pruritic rash; Itching |
| High Dose | INSULIN (HUMAN) | Hypoglycemia; Confusion; Fall/  Hypoglycemia | Drug-induced hypoglycemia | Hypoglycemia |
| Adverse Drug Reaction | EPINEPHRINE | Headache | No Match | Migraine aura without headache |
| Adverse Drug Reaction | WARFARIN | Hematoma | Abdominal discomfort | Abdominal distension |
| Needs Additional Drug/Untreated Indication | PROGESTERONE | Miscarriage | Complete abortion | No match |
| Non-Adherence | PHENYTOIN | Seizure | Tonic-clonic seizure | Seizure |
| Needs Additional Drug/Untreated Indication | WARFARIN | Headache | No Match | Migraine aura without headache |
| Non-Adherence | HALOPERIDOL | Rigid; Stiff | Spasticity | No match |
| High Dose | MORPHINE | Fall/  Fracture | Drowsy | No match |
| Needs Additional Drug/Untreated Indication | ACETYLSALICYLIC ACID | Slurred speech | Dysarthria | Disturbance in speech |
| Non-Adherence | INSULIN (HUMAN) | Fall; Hypoglycemia/  Hypoglycemia | Drug-induced hypoglycemia | Hypoglycemia |
| High Dose | IINSULIN (HUMAN) | Hypoglycemia; Confusion; Fall/  Hypoglycemia | Drug-induced hypoglycemia | Hypoglycemia |

**ICD-11**

| **ADE Type** | **ADE Drug(s)** | **ADE Symptoms/**  **Diagnoses** | **ICD RA1** | **ICD RA2** |
| --- | --- | --- | --- | --- |
| Non-Adherence | HYDROCHLORO-THIAZIDE | Weakness/  Stroke | Lacunar syndromes, unspecified; Cerebral ischaemic stroke, unspecified; Cerebral ischaemic stroke due to small artery occlusion; Disorders of muscles, unspecified | Transient ischaemic attack, unspecified |
| Non-Adherence | INSULIN (HUMAN) | Hyperglycemia | Other specified elevated blood glucose level | Diabetes mellitus, type unspecified |
| Low Dose | CANDESARTAN | Hypertension | Isolated systolic hypertension | Combined diastolic and systolic hypertension; Vertigo, unspecified |
| Non-Adherence | LEVONORGESTREL  ESTRADIOL | Bleeding; Menstruation/  Dysmenorrhea | Haemorrhage, not elsewhere classified; Other specified abnormal uterine or vaginal bleeding | Dysmenorrhoea; Heavy menstrual bleeding |
| Low Dose | WARFARIN | INR subtherapeutic/  Lab abnormality | Other specified abnormal laboratory results, not elsewhere classified | Abnormal laboratory results, not elsewhere classified, unspecified |
| Adverse Drug Reaction | HYDROCORTISONE  PROCTOSONE | Rash; Itchiness/  Allergic reaction | Allergic or hypersensitivity disorders involving skin or mucous membranes; Rash, unspecified; Drug-induced pruritus; Pruritus, unspecified | Drug-induced urticaria; Itching of skin; |
| Ineffective Drug | CEFALEXIN | Worsening foot infection/  Cellulitis | Staphlyococcal cellulitis of skin; Methicillin resistant Staphylococcus aureus; Cutaneous involvement by other specified bacterial infection; Cellulitis disorder (TM1) | Bacterial cellulitis or lymphangitis due to unspecified bacterium |
| Adverse Drug Reaction | CLOPIDOGREL | Eye and hand swelling/  Allergic reaction | Allergic or hypersensitivity disorders involving skin or mucous membranes; Drug-induced angioedema; Oedema of orbit | Drug hypersensitivity of unspecified type |
| Adverse Drug Reaction | NAPROXEN | Epigastric pain | Pain, unspecified | Nausea; Pain localised to upper abdomen |
| Adverse Drug Reaction | HEPATITIS A INACTIVATED WHOLE VIRUS | Tongue swelling/  Allergic reaction | Symptom or complaint of the mouth, tongue or lip; Lipoedema; Drug-induced angioedema | Allergic or hypersensitivity conditions of unspecified type |
| Needs Additional Drug/Untreated Indication | ACETAMINOPHEN  CODEINE  FLUOXETINE  CAFFEINE  OXYCODONE | Medication required | Inappropriate stoppage or discontinuation of drug without injury or harm | Depression disorder (TM1) |
| Drug Interaction | CANDESARTAN  FUROSEMIDE  CONTRAST MEDIA | Creatinine elevated/  Lab abnormality | Other specified abnormal laboratory results, not elsewhere classified | Abnormal laboratory results, not elsewhere classified, unspecified |
| Adverse Drug Reaction | CANDESARTAN  BISOPROLOL  ROSUVASTATIN | Congested ears; Throat scratchy; Swollen external neck | Other specified diseases of the ear or mastoid process; Other specified eye, ear, nose and throat system disorders (TM1); Other specified symptom or complaint of the throat; Symptom or complaint of the neck | Nasal congestion; Abrasion of throat |
| Needs Additional Drug/Untreated Indication | MORPHINE | Weakness/  Drug withdrawal syndromes | Opioid withdrawal; Disorders of muscles, unspecified | Ataxia, unspecified; MDMA or related drug withdrawal, including MDA; Other specified psychoactive substance withdrawal, unspecified |
| Adverse Drug Reaction | PERINDOPRIL | Creatinine elevated | Acute kidney failure, stage unspecified; Other specified abnormal laboratory results, not elsewhere classified | Disorders of creatine metabolism; Abnormal laboratory results, not elsewhere classified, unspecified |
| Adverse Drug Reaction | GEMCITABINE | Pancytopenia | Drug-induced cytopenia | Aplastic anaemia, unspecified |
| Adverse Drug Reaction | PROGUANIL COMBINATIONS  ATOVAQUONE | Hepatitis | Drug-induced or toxic liver disease with acute hepatic necrosis or acute hepatitis | Acute viral hepatitis, unspecified |
| Adverse Drug Reaction | NSAIDS | Medication overuse headache/  Headache | Other specified headache disorders | Headache disorders, unspecified; Migraine, unspecified |
| Adverse Drug Reaction | CONTRAST MEDIA | Anaphylactoid reaction/  Allergic reaction | Drug-induced anaphylaxis | Anaphylaxis, unspecified |
| High Dose | WARFARIN | Abnormal lab values; Elevated INR/  Lab abnormality | Other specified abnormal laboratory results, not elsewhere classified | Abnormal laboratory results, not elsewhere classified, unspecified |
| Non-Adherence | PHENYTOIN | Seizure | Generalised tonic-clonic seizure | Seizure due to other acute cause |
| Non-Adherence | LITHIUM  QUETIAPINE | Abnormal behaviour/  Mania | Odd or peculiar behaviour; Manic disorder (TM1) | Disorganized behaviour; Manic symptoms in primary psychotic disorders |
| Non-Adherence | RISPERIDONE | Refusal to take meds or leave home/  Schizophrenia | Personal history of noncompliance with medical treatment or regimen | Schizophrenia, unspecified |
| Non-Adherence | LEVOTHYROXINE SODIUM | Abnormal lab values/  Hyperthyroidism | Disorders of the thyroid gland or thyroid hormones system, unspecified; Abnormal results of thyroid function studies | Abnormal laboratory results, not elsewhere classified, unspecified; Secondary hyperthyroidism |
| Low Dose | HYDROMORPHONE | Pain/  Infection | Acute serous or mucoid otitis media; Pain, unspecified; Otalgia; Vertigo, unspecified | Other specified acute pain; Otitis media, unspecified |
| Needs Additional Drug/Untreated Indication | HYDROMORPHONE | Abdominal pain | Other and unspecified abdominal pain | Generalised abdominal pain |
| Ineffective Drug | TRAMADOL | Abdominal pain/  Hernia | Other and unspecified abdominal pain; Other specified hernias | Generalised abdominal pain; Other specified intra-abdominal hernia |
| Non-Adherence | ATAZANVIR  RITONAVIR  TRUVADA | Abnormal lab values; Odynophagia | Other specified abnormal laboratory results, not elsewhere classified; Dysphagia | Abnormal laboratory results, not elsewhere classified, unspecified; Dysphasia |
| Adverse Drug Reaction | HYDROCHLORO-THIAZIDE | Pain/  Gout | Drug-induced gout; Symptom or complaint of the ankle | Pain, unspecified; Gout, unspecified |
| Drug Interaction | QUETIAPINE  AMITRIPTYLINE  BACLOFEN  CLONAZEPAM | Slurred speech; Unable to ambulate | Speech disturbances, unspecified; Dysarthria, unspecified; Immobility; Clouding of consciousness | Other specified symptoms or signs involving speech or voice; Loss of consciousness, duration unspecified or unknown due to lack of information |
| Adverse Drug Reaction | ZOPICLONE | Weakness; Dizziness/  Falls | Other specified disorders of muscles; Syncope and collapse, unspecified | Dizziness and giddiness, unspecified; Exposure to fall on the same level or from less than 1 metre |
| Needs Additional Drug/Untreated Indication | WARFARIN | Confusion/  Stroke | Disorientation; Cerebral ischaemic stroke, unspecified | Confused, disoriented speech; Stroke not know if ischaemic or haemorrhagic |
| Drug Interaction | DIPHENHYDRAMINE  HYDROMORPHONE  MORPHINE | Urinary retention | Retention of urine | Urinary symptom or complaint |
| Adverse Drug Reaction | ACETYLSALICYCLIC ACID | Anemia | Anaemias or other erythrocyte disorders, unspecified | No match |
| Non-Adherence | ARIPIPRAZOLE | Increased energy; Impulsivity/  Mania | Bipolar type I disorder, current episode manic, with psychotic symptoms | Manic symptoms in primary psychotic disorders |
| Low Dose | WARFARIN | Abnormal lab values; Swelling/  Deep vein thrombosis | Lower limb deep vein thrombosis; Other specified abnormal laboratory results, not elsewhere classified | Abnormal laboratory results, not elsewhere classified, unspecified; Other specified deep vein thrombosis |
| Ineffective Drug | OXYCODONE  CYCLOBENZAPRINE | Pain/  Chronic pain | Pain, unspecified | Chronic pain, unspecified |
| High Dose | LITHIUM | Weakness; Creatinine elevated; Hypotension | Other specified disorders of muscles; Orthostatic hypotension; Kidney failure, unspecified | Unspecified myasthenia gravis or neuromuscular junction disorders; Hypotension, unspecified; Abnormal laboratory results, not elsewhere classified, unspecified |
| Non-Adherence | BUPROPION | Anxiety; Insomnia | Anxiety or fear-related disorders, unspecified; Insomnia disorders, unspecified | Anxiety; Short-term insomnia |
| Needs Additional Drug/Untreated Indication | CODEINE | Pain; Med request/  Chronic pain | Pain, unspecified | Chronic pain, unspecified |
| Ineffective Drug | ERYTHROMYCIN | Chest pain; Cough/  Pneumonia | Dyspnoea; Pneumonia, organism unspecified | Chest pain, unspecified; Other specified pneumonia; Cough |
| Non-Adherence | INSULIN (HUMAN) | Hyperglycemia; General unwell | Diabetic ketoacidosis without coma | Diabetic hyperosmolar hyperglycaemic state, unspecified |
| Adverse Drug Reaction | PREDNISONE | Hyperglycemia; Confusion; Nausea; Lightheadedness/  Diabetes | Other specified elevated blood glucose level; Disorientation; Polyuria | Diabetic hyperosmolar hyperglycaemic state, unspecified; Confused, disoriented speech; Nausea |
| Adverse Drug Reaction | HYROCHLORO-THIAZIDE | Seizure/  Lab abnormality | Other specified epilepsy or seizures; Hypo-osmolality or hyponatraemia | Type of seizure, unspecified; Abnormal laboratory results, not elsewhere classified, unspecified |
| Adverse Drug Reaction | GENTAMICIN  CIPROFLOXACIN | Thrush | Candidiosis of lips or oral mucous membranes | Thrush disorder (TM1) |
| Needs Additional Drug/Untreated Indication | VALPROIC ACID | Abnormal behaviour; Grandiose disorganized behaviour/  Mania | Manic symptoms in primary psychotic disorders | Bipolar type I disorder, current episode manic, with psychotic symptoms |
| Needs Additional Drug/Untreated Indication | WARFARIN | Slurred speech; Facial asymmetry/  Stroke | Cerebral ischaemic stroke due to cardiac embolism; Dysarthria, unspecified | Stroke not known if ischaemic or haemorrhagic; Facial asymmetry |
| High Dose | PHENYTOIN | Fall; Abnormal lab values/  Falls | Unintentional fall from unspecified height; Other specified abnormal laboratory results, not elsewhere classified | Abnormal laboratory results, not elsewhere classified, unspecified; Exposure to fall on the same level or from less than 1 metre |
| Transcription/ Dispensing/ Administration Error | BISOPROLOL  LEVOTHYROXINE SODIUM | Error | Other specified mode of injury or harm associated with exposure to a drug, medicament | No match |
| Adverse Drug Reaction | CIPROFLOXACIN | Rash; Periorbital edema/  Allergic reaction | Drug-induced pruritus; Allergic or hypersensitivity disorders involving the eye; Allergic or hypersensitivity disorders involving skin or mucous membranes | Allergic or hypersensitivity conditions of unspecified type; Oedema of eyelid; Drug-induced urticaria |
| Ineffective Drug | CLINDAMYCIN | Purulence; Foul smell/  Infection | Other specified surgical site infection | Post traumatic wound infection, not elsewhere classified |
| Non-Adherence | ACETYLSALICYCLIC ACID  CLOPIDOGREL  ATORVASTATIN  ATENOLOL  RAMIPRIL | Chest pain; Abnormal lab values/  Acute coronary syndrome | Acute ischaemic heart disease, unspecified | Chest pain, unspecified; Other specified diseases of coronary artery |
| Needs Additional Drug/Untreated Indication | AMLODIPINE | Hypertension; Troponin increase/  Hypertension | Combined diastolic and systolic hypertension | Essential hypertension, unspecified; Other specified abnormal laboratory results, not elsewhere classified |
| Low Dose | WARFARIN | INR subtherapeutic/  Pulmonary embolism | Pulmonary thromboembolism, unspecified; Other specified abnormal laboratory results, not elsewhere classified | Pulmonary embolism as current complication following acute myocardial infarction; Abnormal laboratory results, not elsewhere classified, unspecified |
| Drug Interaction | QUETIAPINE  HYDROMORPHONE  ZOPICLONE | Sedation | Somnolence, not elsewhere classified | Sedative |
| Drug Withdrawal | CLONAZEPAM | Anxiety; Agitated; Confused; Delusional/  Drug withdrawal syndromes | Sedative, hypnotic or anxiolytic withdrawal, unspecified; Anxiety or fear-related disorders, unspecified; Psychomotor agitation | Other specified psychoactive substance withdrawal, unspecified; Anxiety; Delusion, unspecified |
| Non-Adherence | METHIMAZOLE | Anxiety; Abnormal lab values | Anxiety or fear-related disorders, unspecified; Other specified disorders of the thyroid gland or thyroid hormones system | Anxiety; Secondary hyperthyroidism; Other specified abnormal laboratory results, not elsewhere classified |
| Drug Withdrawal | ZOPICLONE | Hallucination visual | Visual hallucinations | Visual release hallucination |
| Adverse Drug Reaction | AMOXICILLIN | Rash; Throat swelling/  Allergic reaction | Other specified allergic or hypersensitivity disorders involving the respiratory tract; Allergic or hypersensitivity disorders involving skin or mucous membranes | Allergic or hypersensitivity conditions of unspecified type; Rash generalised |
| Non-Adherence | OLANZAPINE | Abnormal behaviour; Confusion/  Psychosis | Acute and transient psychotic disorder, unspecified; Disorientation | Schizophrenia or other primary psychotic disorders, unspecified; Confused, disoriented speech |
| Adverse Drug Reaction | WARFARIN | Bright red blood per rectum | Haematochezia | No match |
| Low Dose | FUROSEMIDE | Edema; Swelling of feet and forearms/  Edema | Other specified generalised oedema | Oedema of legs |
| Adverse Drug Reaction | IRON DEXTRAN | Rash; Hives/  Allergic reaction | Drug-induced urticaria; Allergic or hypersensitivity disorders involving skin or mucous membranes | Allergic or hypersensitivity conditions of unspecified type; Rash, unspecified |
| Non-Adherence | WARFARIN | INR supratherapeutic; Blood in stool/  Lab abnormality | Melaena; Abnormal coagulation profile | Other specified abnormal laboratory results, not elsewhere classified; Positive occult blood in stool |
| Adverse Drug Reaction | CONTRAST MEDIA | Hypotension; Urticaria; Rigor/  Allergic reaction | Drug-induced urticaria; Chills | Allergic or hypersensitivity conditions of unspecified type; Hypotension, unspecified |
| Adverse Drug Reaction | IRON PREPARATIONS ORAL | Shortness of breath; Tachycardia; Throat swelling/  Allergic reaction | Allergic or hypersensitivity disorders involving the respiratory tract, unspecified; Other specified allergic or hypersensitivity disorders involving the respiratory tract | Allergic or hypersensitivity conditions of unspecified type; Dyspnoea; Tachycardia, unspecified |
| Adverse Drug Reaction | RITUXIMAB  FLUDARABINE | Anemia; Tired/  Anemia | Anaemias or other erythrocyte disorders, unspecified | Other specified abnormality of red blood cells |
| Needs Additional Drug/Untreated Indication | ACETYLSALICYLIC ACID | Word finding difficulties | Aphasia | Transient ischaemic attack, unspecified |
| High Dose | VORICONAZOLE | Delirium | Delirium due to unknown or unspecified aetiological factors | Opioid-induced delirium |
| High Dose | HYDROMORPHONE | Diaphoresis; Tremor | Secondary tremor, unspecified; Hyperhidrosis, unspecified | Complaint of abnormal sweating; Tremor due to chronic or acute substance use |
| High Dose | BROMOCRIPTINE | EPS | Abnormal involuntary movements, unspecified | No match |
| Adverse Drug Reaction | ALLERGY SHOT | Shortness of breath; Chest tightness/  Allergic reaction | Allergic or hypersensitivity disorders involving the respiratory tract, unspecified | Dyspnoea; Chest pain, unspecified; Allergic or hypersensitivity conditions of unspecified type |
| Adverse Drug Reaction | DILTIAZEM  TIMOLOL | Other | Complete atrioventricular block, unspecified | Bradycardia, unspecified |
| Adverse Drug Reaction | AMOXICILLIN | Shortness of breath; Itchiness/  Allergic reaction | Other specified allergic or hypersensitivity disorders involving the respiratory tract; Drug-induced pruritus | Allergic or hypersensitivity conditions of unspecified type; Dyspnoea |
| High Dose | WARFARIN | INR supratherapeutic; Hematuria/  Lab abnormality | Haematuria, unspecified | Other specified abnormal laboratory results, not elsewhere classified |
| Drug Interaction | WARFARIN  PRASUGREL  ACETYLSALICYLIC ACID | Rectal bleed; Blood in stool; INR supratherapeutic | Haematochezia | Positive occult blood in stool; Other specified abnormal laboratory results, not elsewhere classified |
| Non-Adherence | ZOPICLONE | Insomnia | Insomnia disorder (TM1) | Short-term insomnia |
| Adverse Drug Reaction | FAMPRIDINE | Tremors | Other specified secondary tremor | Secondary tremor, unspecified |
| Drug Interaction | WARFARIN  CLARITHROMYCIN | Hematuria;  INR supra-therapeutic/  Bleed (not GI) | Haematuria, unspecified | Other specified abnormal laboratory results, not elsewhere classified |
| Needs Additional Drug/Untreated Indication | INSULIN (HUMAN) | Med request/  Diabetes | Other specified elevated blood glucose level | Diabetic hyperosmolar hyperglycaemic state, unspecified |
| High Dose | CLOBAZAM | Weakness | Other specified disorders of muscles | Dissociative neurological symptom disorder, with paresis or weakness; Unspecified myasthenia gravis or neuromuscular junction disorders |
| Adverse Drug Reaction | ITRACONAZOLE | Rash; Angioedema/  Allergic reaction | Allergic or hypersensitivity disorders involving skin or mucous membranes; Rash, unspecified; | Allergic or hypersensitivity conditions of unspecified type; Rash generalised |
| Non-Adherence | PHENYTOIN | Seizure | Generalised tonic-clonic seizure | Type of seizure, unspecified |
| Non-Adherence | HALOPERIDOL | Rigid; Stiff | Other specified disorders of muscles | Stiffness of joint; Abdominal rigidity |
| High Dose | MORPHINE | Fall/  Fracture | Unintentional fall from unspecified height; Somnolence, not elsewhere classified | Fracture of neck, unspecified; Exposure to fall on the same level or from less than 1 metre |
| Needs Additional Drug/Untreated Indication | WARFARIN | Leg pain/  Deep vein thrombosis | Lower limb deep vein thrombosis | Other specified deep vein thrombosis |
| Needs Additional Drug/Untreated Indication | ACETYLSALICYLIC ACID | Slurred speech | Dysarthria, unspecified | Speech disturbances, unspecified |
| Drug Interaction | IBUPROFEN  FUROSEMIDE  METOLAZONE  SPIRONOLACTONE | Syncope/  Acute kidney injury | Acute kidney failure, stage unspecified; Syncope disorder (TM1) | Syncope and collapse, unspecified; Other specified acute kidney failure |
| Adverse Drug Reaction | GENTAMICIN  CIPROFLOXACIN | Thrush | Candidosis of lips or oral mucous membranes | Thrush disorder (TM1) |
| Drug Interaction | VENLAFAXINE  PRIMIDONE  MIRTAZAPINE  METOCLOPRAMIDE | Delirium; Tremor; Weakness; Diarrhea | Sedative, hypnotic or anxiolytic-induced delirium; Essential tremor or related tremors; Disorders of muscles, unspecified; Diarrhoea | Delirium induced by other specified psychoactive substance including medications |
